# Should we assume that hypothermia is a dysfunction in sepsis?

**DOI:** 10.1186/s13054-016-1584-y

**Published:** 2017-01-11

**Authors:** Alexandre A. Steiner, Monique T. Fonseca, Francisco G. Soriano

**Affiliations:** 1 Department of Immunology, Institute of Biomedical Sciences, University of São Paulo, Avenida Professor Lineu Prestes 1730, São Paulo, SP 05508-000 Brazil; 2 Department of Emergency Medicine, Medical School, University of São Paulo, Avenida Doutor Arnaldo 455, São Paulo, SP 01246-903 Brazil

Letter

Wiewel et al. [1] clearly showed that development of hypothermia instead of fever in sepsis is not tied to a switch from a pro-inflammatory to an anti-inflammatory state. The authors then suggest that vascular dysfunction could play a role in hypothermia. While this hypothesis deserves attention, we urge researchers to consider that there is no hard evidence indicating that hypothermia is a dysfunction in sepsis.

Not all systems fail simultaneously in sepsis, and those with preserved function are likely to launch evolutionarily conserved compensatory responses. Could thermoregulation be preserved during septic hypothermia? Could hypothermia be adaptive when the costs of fever exceed its benefits? According to evidence from rat models of systemic inflammation, the answers to these questions may be yes. First, hypothermia in endotoxemic rats is an early, transient phenomenon that is not consequential to circulatory shock [2]. Second, hypothermia in endotoxic shock is brought about by downregulation of thermogenesis when thermogenic capacity is unimpaired [2, 3]. Third, rats with endotoxic shock do not attempt to restore normothermia when given the chance to select a warmer environment; on the contrary, they seek a cooler environment [3]. Last, spontaneous hypothermia has been shown to be more advantageous than fever in rats with severe forms of endotoxemia and *Escherichia coli* sepsis [2, 4].

There has been a complete disconnect between these experimental data and clinical studies on this subject. Recently, though, Fonseca et al. [5] published the first effort to reconcile experimental and clinical evidence on septic hypothermia. That study revealed that, similarly to animal models of endotoxemia, hypothermia in human sepsis is usually self-limiting and transient. Perhaps most importantly, hypothermia was rarely observed in the moments that preceded death, when multiple organ failure is presumably at its peak. Hence, it is possible that an early, regulated form of hypothermia exists in human sepsis. By the same token, the reported association between hypothermia and higher mortality should not be taken as evidence that hypothermia is a dysfunction that worsens sepsis. This association could merely reflect the fact that hypothermia replaces fever in the most severe cases of sepsis, both in rats and humans. In our opinion, the impact of septic hypothermia on clinical outcomes can only be adequately addressed by an interventional study in which spontaneous hypothermia is allowed or prevented within the hypothermic subset of septic patients. We are planning such a study and invite those interested to join us.
