# Supplementary material for: Should we assume that hypothermia is a dysfunction in sepsis?
Source: Crit Care. 2017 Jan 11;21:8. doi: 10.1186/s13054-016-1584-y (PMC5225576; doi:10.1186/s13054-016-1584-y)
Supplement: Related texts

# Reply: Authors’ response

**Authors:** Matthew B. Harmon, Maryse A. Wiewel, W. Joost Wiersinga, Nicole P. Juffermans

We are thankful for the letter of Steiner and colleagues in response to our paper on risk factors, host response and outcome of hypothermic sepsis [1]. We fully acknowledge the authors’ contributions to the field and their efforts to reconcile experimental and clinical evidence on septic hypothermia [5].

We agree with the authors that hypothermia could be an adaptive response during sepsis. It may be hypothesized that once the metabolic cost of fever outweighs its immune stimulatory benefits, the host may become hypothermic, thereby decreasing metabolism and also potentially decreasing inflammation. We also agree that our study was not designed to provide definitive evidence that hypothermia is a dysfunction in sepsis. As mentioned in the limitation section, our study was observational and cause–effect relationships cannot be established due to the nature of the study design. Indeed, findings which have been associated with hypothermia in previous studies, such as increased lymphopenia [6] and increased levels of fractalkine [1], can also be linked to increased disease severity and not to hypothermia per se.

Some of the experimental work may relate to clinical findings. Spontaneous hypothermia in rat endotoxemia may be a pre-emptive strategy to prevent hypoxia [2]. In comparison, patients who are more prone to hypoxia or a metabolic deficit may also develop hypothermia more often, such as those with preexisting circulatory dysfunction (i.e., chronic cardiovascular dysfunction) or those with few metabolic reserves (i.e., low body mass index). That said, however, it is difficult to reconcile an adaptive response in rodents to an evidently increased mortality noted in observational studies in patients [1, 6].

A remark on the interpretation of findings in experimental models is that regulation of body temperature in rodents is profoundly different than in humans due to differences in the ratio of body content to body surface [7]. Therefore, experimental results need to be validated in clinical studies. We look forward to the results of an interventional study in which spontaneous hypothermia is allowed or prevented within the hypothermic subset of patients with sepsis. We would like to participate in this effort and we suggest that this trial includes analyses on the host response, including markers of immune suppression and endothelial dysfunction, to provide further insight into the etiology of hypothermia in sepsis pathogenesis.
